# Editorial: Recent advances in big data, machine, and deep learning for precision agriculture

**DOI:** 10.3389/fpls.2024.1367538

**Published:** 2024-02-09

**Authors:** Marcin Woźniak, Muhammad Fazal Ijaz

**Affiliations:** ^1^ Faculty of Applied Mathematics, Silesian University of Technology, Gliwice, Poland; ^2^ School of Engineering and Information Technology, Melbourne Institute of Technology, Melbourne, VIC, Australia

**Keywords:** precision agriculture (PA), big data & analytics, machine learning, plant disease, technology

Precision agriculture involves the use of real-time information to enhance the efficient use of resources and the oversight of farming methods, all while minimizing adverse environmental effects. Thanks to advancements in remote sensing technologies, there is now a substantial amount of big data being produced within the agricultural sector. This data, which needs to be transformed into valuable information, has proven to be beneficial when subjected to analysis using machine and deep learning techniques applied to remote sensing products. This Research Topic “*Recent advances in big data, machine, and deep learning for precision agriculture*” has attracted 20 high quality articles that cover the state-of-the-art applications and technical development of artificial intelligence, big data, features optimization, crop disease detection and classification for precision agriculture. In the ever-evolving landscape of agriculture, three pivotal themes have emerged as beacons of transformative change. This editorial delves into the realms of innovation that are shaping the future of agriculture, focusing on three interconnected themes: advances in plant disease detection and crop health monitoring, integration of Artificial Intelligence (AI) and Machine Learning (ML) in precision agriculture, and innovative approaches for crop production optimization.

In the realm of agricultural sciences, the dynamic landscape of plant disease detection and crop health monitoring has witnessed substantial progress, thanks to pioneering research endeavors. Shoaib et al. confronted the persistent challenge of manually monitoring plant diseases by underscoring the pivotal role of machine learning technology. Their work proposed a sophisticated deep learning-based system, leveraging a convolutional neural network (Inception Net) trained on a substantial dataset comprising 18,161 segmented and non-segmented tomato leaf images. Noteworthy were the employment of two state-of-the-art semantic segmentation models, U-Net and Modified U-Net, for disease detection and segmentation. The outcomes showcased the Modified U-Net model’s superior performance, surpassing existing methods and affirming its efficacy in classifying plant diseases with high accuracy. Addressing the global issue of Bacteriosis infections in peach crops, Akbar et al. presented a groundbreaking solution employing a novel Lightweight Convolutional Neural Network (WLNet) based on the Visual Geometry Group (VGG-19). Their study incorporated a dataset of 10,000 images, preprocessed through various techniques, to train the proposed model for detecting and classifying Bacteriosis and healthy peach leaves. Remarkably, the LWNet model achieved an impressive accuracy of 99%, outperforming established models such as LeNet, Alexnet, VGG-16, and the simple VGG-19. This underscored the effectiveness of the LWNet model in Bacteriosis detection.

In a parallel effort, Shoaib et al. presented a context-aware 3D CNN model for lesion segmentation and a Deep CNN for lesion subtype recognition, followed by survival prediction using a hybrid CNN and Linear Regression approach. Evaluating their model on the Plant Village Benchmark Dataset, the segmentation model attained an accuracy of 92%, while the subtype recognition model exhibited high accuracies across various plants, suggesting its potential for real-time disease detection and crop health monitoring. Lamba et al. delved into the impact of paddy leaf diseases on grain production, emphasizing the imperative for accurate detection. Their study utilized a dataset of 4,068 paddy leaf images, amalgamating primary and secondary data from repositories like Mendeley and Kaggle. Employing a generative adversarial network (GAN), they augmented the dataset and proposed a hybrid approach involving a convolutional neural network (CNN) and support vector machine (SVM) for disease severity classification. Impressively, the model achieved a 98.43% accuracy in predicting bacterial blight, blast, and leaf smut infections, outperforming existing CNN and SVM models. Salman et al. explored the application of deep learning in plant disease detection for precision agriculture. Their comprehensive overview discussed the pros and cons of existing approaches, highlighting advancements in machine-learning-based disease detection, prevalent datasets, and emerging techniques. The paper aimed to inform researchers and practitioners in the field, inspiring future research efforts for enhanced crop health management. Batin et al. introduced SPIKE-segm, a curated dataset, and WheatSpikeNet, an optimized instance segmentation approach, to accurately count wheat spikes in field imagery. Their proposed method, based on Cascade Mask RCNN with enhancements and hyperparameter tuning, demonstrated superior detection and segmentation performance, outperforming existing state-of-the-art methods and showing potential for precise yield estimation in wheat plants. Wei et al. put forth a multi-scale fusion Recurrent Feature Reasoning (RFR) network to enhance accuracy in grading potted anthurium plants through machine vision. The network, equipped with a multi-layer component in the feature reasoning module, effectively addressed occlusion problems during image capture. Comparative experiments showcased the superiority of the proposed network over traditional image completion models, particularly excelling in handling large-area incomplete images. In another noteworthy contribution, Shoaib et al. introduced the EG-CNN model, a novel explainable gradient-based approach for predicting plant diseases using omics data and hyperspectral images. Their study demonstrated the model’s resilience to hyperparameter variations, faster testing times compared to baseline models, and effective capture of crucial aspects of plant diseases through a qualitative analysis using saliency maps. The research emphasized the potential of deep learning in plant bioinformatics for accurate and robust disease detection. The collective impact of these groundbreaking studies not only addresses the current challenges in plant disease detection but also opens avenues for further exploration and innovation in the realm of precision agriculture and crop health management.

In the rapidly evolving landscape of precision agriculture, the integration of AI and ML has emerged as a game-changer. The exploration by Mesías-Ruiz et al. delves into the pivotal role that AI and ML play in addressing the challenges of crop protection, particularly in the face of climate change and escalating pest incidences. The authors trace the evolution of crop protection from precision agriculture (Ag1.0) to the present mature stage aligned with Ag5.0, highlighting the integration of ML and cutting-edge agricultural technologies for precision crop protection. A comprehensive taxonomy of ML algorithms is presented, accompanied by a bibliometric study on over 120 algorithms. The scientific impact of ML, with a specific focus on its application in the detection and control of crop diseases, is thoroughly analyzed. Furthermore, the article outlines 39 emerging technologies, including smart sensors and AI-based robotics, that are shaping the future of digitized, smart, and real-time crop protection in Ag5.0. The concluding section succinctly summarizes key insights and remarks, providing a comprehensive view of the transformative potential of AI and ML in modern agriculture.

Shoaib et al. delves into recent advancements (2015-2022) in the application of ML and DL techniques for plant disease identification. They highlight challenges and limitations associated with ML and DL in plant disease identification, such as issues related to data availability, imaging quality, and distinguishing between healthy and diseased plants. By offering solutions to these challenges and limitations, this research provides valuable insights for researchers, practitioners, and industry professionals involved in plant disease detection. It presents a comprehensive understanding of the current state of research in this field, outlining both the benefits and limitations of ML and DL methods.

In a related study, Zhou et al. investigate the impact of parameters such as canopy height, fractional vegetation cover, and spectral indices on the accuracy of yield prediction. Their results underscore the significance of integrating agronomic trait parameters with spectral features, showcasing substantial improvements in prediction accuracy. The study identifies a potent combination of canopy height, fractional vegetation cover, normalized difference red-edge index, and enhanced vegetation index that yields the best results. Notably, the research reveals enhanced prediction accuracies during specific growth stages, particularly heading, and across multiple stages compared to single stages. Despite weaker predictions across different cultivars, the combination of agronomic traits and spectral indices proves instrumental in improving predictions. Singh et al. shift the focus to the implementation of IoT-based smart farming for growing tomatoes. Emphasizing ultrafast response and efficiency, the authors present a smart framework employing IoT devices for monitoring and automating tasks such as moisture prediction, irrigation, and nutrient levels. Large-scale experiments validate the model’s effectiveness in intelligently monitoring the irrigation system, contributing to higher tomato yields. Additionally, a forthcoming smartphone app is poised to provide farmers with essential data on the health of their tomato crops, exemplifying the practical applications of AI and ML in real-time farming solutions.

In the realm of image acquisition and processing, Jin et al. introduce EBG_YOLOv5, an optimized version of YOLOv5 with enhanced precision and reduced computational requirements. Ablation and comparison experiments showcase EBG_YOLOv5’s superiority over its predecessor, demonstrating higher precision, recall rates, and mAP0.5 while simultaneously reducing model size. The improved model exhibits superior accuracy and a smaller size, paving the way for its application in hydroponic lettuce defective leaf detection. Tan et al. contribute a novel method, Im-YOLOv5s, for navigation line extraction in complex agricultural environments. Utilizing deep learning and least squares algorithms, the authors enhance the YOLOv5s algorithm, achieving higher detection performance with notable improvements in accuracy and frame rate. The results demonstrate the effective detection of complex agricultural features, meeting the requirements for intelligent mechanization in Panax notoginseng planting.

On the other hand, M. Abdullah et al. tackle challenges in plant disease detection by addressing issues related to data availability, class imbalance, and data augmentation. Their study compares the performance of diffusion-based model RePaint with state-of-the-art GAN model InstaGAN, showcasing the superior quantitative results of RePaint. The study highlights the potential of diffusion models in data augmentation for plant disease detection, opening new avenues for research in this domain. Bi et al. propose a transformer-based approach for segmenting images into plant and soil categories. Employing vision transformer modules for feature extraction and handling time-series features, the method exhibits a remarkable reduction in prediction error compared to baseline models. The study sheds light on the crucial impact of seed information on predictions, particularly for low yields, demonstrating the versatility and effectiveness of transformer-based approaches in precision agriculture. These groundbreaking studies collectively underscore the transformative power of AI and ML in advancing precision agriculture. From crop protection to yield prediction, smart farming, image processing, and disease detection, these innovative approaches mark a paradigm shift in the way we cultivate and manage crops, heralding a new era of efficiency, sustainability, and intelligence in agriculture.

In the realm of agricultural technology, groundbreaking strides are being made to revolutionize crop production optimization. In the year 2022, Wang et al. introduced Cropformer, a cutting-edge deep learning approach designed for accurate and efficient multi-scenario crop classification using remotely sensed data. The methodology employed by Cropformer entails a two-step classification process, beginning with self-supervised pre-training to accumulate a profound understanding of crop growth, followed by fine-tuned supervised classification using labeled time series data. Cropformer’s prowess surpasses existing methods in a myriad of scenarios, including full-season crop classification, in-season crop classification, few-sample crop classification, and model transfer. Noteworthy is its ability to exhibit higher accuracy with fewer samples, showcasing exceptional efficiency during classification. What sets Cropformer apart is its adept utilization of both unlabeled and labeled data to build *a priori* knowledge, enabling the model to learn generalized features for versatile crop classification.

In 2023, Wang et al. addressed the imperative of real-time fruit detection in the Xiaomila pepper harvesting robot. Their solution, the YOLOv7-PD model, integrates YOLOv7-tiny as a transfer learning model, incorporating modifications such as deformable convolution and the SE attention mechanism. The experiments conducted revealed that YOLOv7-PD outperformed other single-stage detection models, achieving a remarkable mean Average Precision of 90.3%. Furthermore, the model demonstrated a reduction in size from 12.7 MB to 12.1 MB and a decrease in computational complexity from 13.1 GFlops to 10.3 GFlops, making it a more effective and computationally efficient solution for Xiaomila fruit detection. Hasan et al. directed their focus towards Bangladesh’s agriculture in 2023, addressing the global challenge of insufficient agricultural growth to meet the increasing demand for food. Through the Research Topic and preprocessing of data from relevant institutions, they proposed an ensemble machine learning approach, K-nearest Neighbor Random Forest Ridge Regression (KRR), to predict major crop production. KRR demonstrated superior performance, exhibiting low mean square error and high R2 values for rice, wheat, and potato production. Additionally, a recommender system was designed to suggest suitable crops for specific land areas, aiming to assist farmers and enhance overall agricultural productivity. In the same year, Hou et al. proposed a groundbreaking occluded cherry tomato recognition model, DSP-YOLOv7-CA, designed to enhance the accuracy of picking robots in natural environments. The model, based on YOLOv7, incorporates null residual edges, depth-separable convolutional layers, jump connections, and a coordinate attention mechanism (CA) to improve feature extraction and attention to occluded tomatoes. The experimental results showcased superior performance, with an impressive 98.86% average detection accuracy, reduced model parameters, and effective recognition of cherry tomatoes even with less than 95% occlusion in real-world scenarios.

Finally, Kao et al. addressed the need for cold-tolerant soybean cultivars, given the crop’s sensitivity to low temperatures. Using an advanced systems biology framework, the researchers identified 44, 143, and 45 cold tolerance genes (CTgenes) for short-, mid-, and long-term cold treatment. These CTgenes outperformed other genes in an independent RNA-seq database and were validated with SNP genotype data, showcasing their effectiveness in distinguishing cold-resistant and cold-sensitive soybean lines. The proposed pipelines offer a robust and efficient solution for biomarker discovery, module discovery, and sample classification in plant traits.

In conclusion, these innovative approaches signify a new era in agricultural technology, promising increased efficiency, accuracy, and sustainability in crop production optimization. As researchers continue to push the boundaries of technological advancements, the agricultural landscape stands to benefit significantly from these groundbreaking developments.

## Author contributions

MW: Project administration, Supervision, Writing – original draft, Writing – review & editing. MI: Project administration, Supervision, Writing – original draft, Writing – review & editing.

